# Exchange Length Tailored Magnetic Resonance for Broadband Absorption in FeCo‐Based Alloys

**DOI:** 10.1002/advs.74207

**Published:** 2026-02-05

**Authors:** Xiaoyang Liu, Yingli Zhu, Shuangxin Zhang, Gangtao Luo, Mengke Qiao, Jiang Wu, Xiangcheng Li

**Affiliations:** ^1^ State Key Laboratory of Advanced Refractories Wuhan University of Science and Technology Wuhan P. R. China; ^2^ Key Laboratory of High Temperature Electromagnetic Materials and Structure of MOE Wuhan University of Science and Technology Wuhan P. R. China

**Keywords:** broadband wave absorption, effective magnetic anisotropy, magnetic exchange length, soft magnetic alloys

## Abstract

The broadband electromagnetic wave absorption (EWA) of soft magnetic alloys (SMAs) is severely constrained by the rapid decay of their magnetic loss at gigahertz frequencies. To address this issue, an intergranular coupling strategy guided by the magnetic exchange length (*L*_ex_) is proposed in this study. Doping with Cu systematically increases *L*_ex_ in SMAs from 12.7 nm to 19.1 nm, thereby enhancing the collective precession of magnetic moments over a larger spatial range. This Cu doping concurrently reduces the effective magnetic anisotropy (*K*_eff_) from 9.2 to 8.6 kJ m^−3^ and increases the magnetic exchange strength by 25%. These coordinated changes effectively shift the resonance peak to 12.0 GHz and amplify the magnetic loss. As a result, the optimized alloy achieves an effective absorption bandwidth (EAB) of 8.1 GHz at a minimal thickness of 1.8 mm, representing a 47.1% improvement over its Cu‐free counterpart. This work pioneers the use of *L*_ex_‐driven mechanisms for regulating magnetic loss and establishes a fundamental framework for enhancing the broadband EWA performance of SMAs through targeted resonance modulation.

## Graphical abstract

To break the high‐frequency magnetic loss bottleneck in soft magnetic alloys, a magnetic exchange length (*L_ex_*)‐guided strategy is proposed. Cu doping increases *L_ex_* and intergranular coupling, which effectively reduces magnetic anisotropy and pushes the exchange resonance to 12.0 GHz. This leads to a remarkably broadened absorption bandwidth of 8.1 GHz at 1.8 mm.

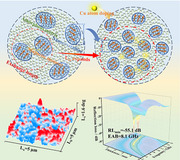

## 1 Introduction

EWA materials are in high demand for applications such as electromagnetic compatibility and wireless communication [1, 2, 3, 4]. As electronic devices evolve toward wider frequency response and integration [5, 6, 7, 8], developing absorber that combines strong absorption, broad bandwidth, and minimal thickness has become a significant frontier challenge [9, 10, 11, 12]. Notably, soft magnetic alloys (e.g., Fe‐Si‐B, Fe‐Co‐based alloys) stand out as ideal candidates for next‐generation absorbing materials offering the merits of high saturation magnetization, excellent permeability, and strong magnetic loss [12, 13, 14, 15]. However, general soft magnetic materials are constrained by the Snoek limit and resonance characteristics [16], facing the dilemma of a relatively narrow EAB and the predicament in modulating the resonance frequency [17, 18].

Component design of soft magnetic alloys enables precise regulation of key electromagnetic parameters [19, 20], such as permeability (*µ*) and magnetic loss tangent (*tanδ_µ_*), making it an effective strategy for optimizing EAB and resonance mechanism. For instance, Duan et al. [21] manipulated the electron spin of magnetic atoms by adjusting the contents of Mn and Al in FeCoNiMnAl, thereby regulating the magnetic moment and increasing the real part of permeability (*µ′*) from 1.8 to 2.3. Luo et al. [22] obtained a *tanδ_µ_* exceeding 0.5 within the 7.0–14.0 GHz range by doping FeCo alloys with rare earth element Sm, this doping induced abundant grain boundaries and facilitated electron transfer between Co and Sm atoms. Additionally, Dou et al. [23] expanded the EAB from 3.5 to 6.2 GHz in FeCoCrAlGd alloys by increasing the Al/Cr ratio, due to the reduction of local compositional segregation and suppression of Cr antiferromagnetism. Component design stimulates the loss mechanisms of materials in different frequency bands, effectively optimizes the key parameters of soft magnetic materials, circumvent the limitations of the traditional Snoek limit. However, broadening the width of the resonance peak still poses significant challenges due to the difficulty in complex correlation between component‐induced microstructural evolution and resonance behavior.

Recent studies have demonstrated that utilizing nanocrystallization techniques to match grain size (*D*) with *L*_ex_ represents a novel research approach to enhance intergranular interactions and magnetic loss mechanism [24]. *L*_ex_ is defined as the critical length scale governing the competition between the exchange interaction (*A*) and *K*_eff_. Within regions smaller than *L*_ex_, the dominant exchange coupling forces magnetic moments to align coherently, thereby suppressing local anisotropy [25, 26]. These alloys form a nanocrystal‐amorphous dual‐phase matrix through heterogeneous nucleation crystallization [19, 27]. Crucially, magnetic domain rotation is hindered at the interface, significantly reducing the magnetic anisotropy constant [25, 26], and consequently expanding the *L*_ex_. When *D* ≤ *L*_ex_, exchange interactions between grains force parallel alignment of all magnetic moments within this range. The resulting magnetic coupling strength exhibits an inverse relationship with grain size, leading to strong magnetic coupling [28, 29], For example, Wang et al. [30] induced nano‐grain orientation along the easy magnetization axis, reducing the grain size. The accompanying 99% increase in *L*_ex_ and the enhancement of exchange stiffness (shown in S6) from 5.2 × 10^−13^ J m^−1^ to 1.8 × 10^−12^ J m^−1^ reflect strengthened magnetic coupling. Cai et al. [28] diminished the grain size from 14 to 12 nm and effectively averaged out the magnetocrystalline anisotropy from 43 to 4 J m^−13^ through composition regulation and magnetic field annealing, facilitating the broadening of the natural resonance peak. Notably, under strong coupling conditions (*D* ≤ *L*_ex_) [31, 32]. While significant progress has been made in optimizing soft magnetic properties, the role of *L*_ex_ in EWA requires deeper exploration. This characteristic is expected to significantly broaden the absorption bandwidth, providing a crucial approach to break through the bottleneck of high‐frequency absorption [33].

Herein, an intergranular coupling strategy guided by *L*_ex_ has been developed. Introducing trace amounts of copper (Cu) into the iron‐cobalt (FeCo) matrix constructs high‐density heterogeneous nucleation sites, leading to grain refinement and a higher density of nanocrystals. The increased grain boundaries resulting from refinement reduce the domain wall energy, broaden the domain width, and thereby realize an increase in *L*_ex_. The regulation of *L*_ex_ aligns the magnetic moments of more grains, reduces the local magnetic anisotropy [34, 35], and enhances the amorphous‐nanocrystal interface coupling [36], improving the exchange stiffness by 25.0%. This mechanism effectively bridges the spectral gap between the low‐frequency natural resonance and the high‐frequency exchange resonance, increases the exchange resonance frequency to 12.0 GHz, and raises the EAB to 8.1 GHz (47.1% higher than that of the Cu‐free sample), with performance superior to most existing studies. In summary, *L*_ex_ regulation technology provides a new direction for the design of soft magnetic alloy‐based EWA materials with excellent absorption performance.

## 2 Elemental Composition and Microstructure

**FIGURE 1:**
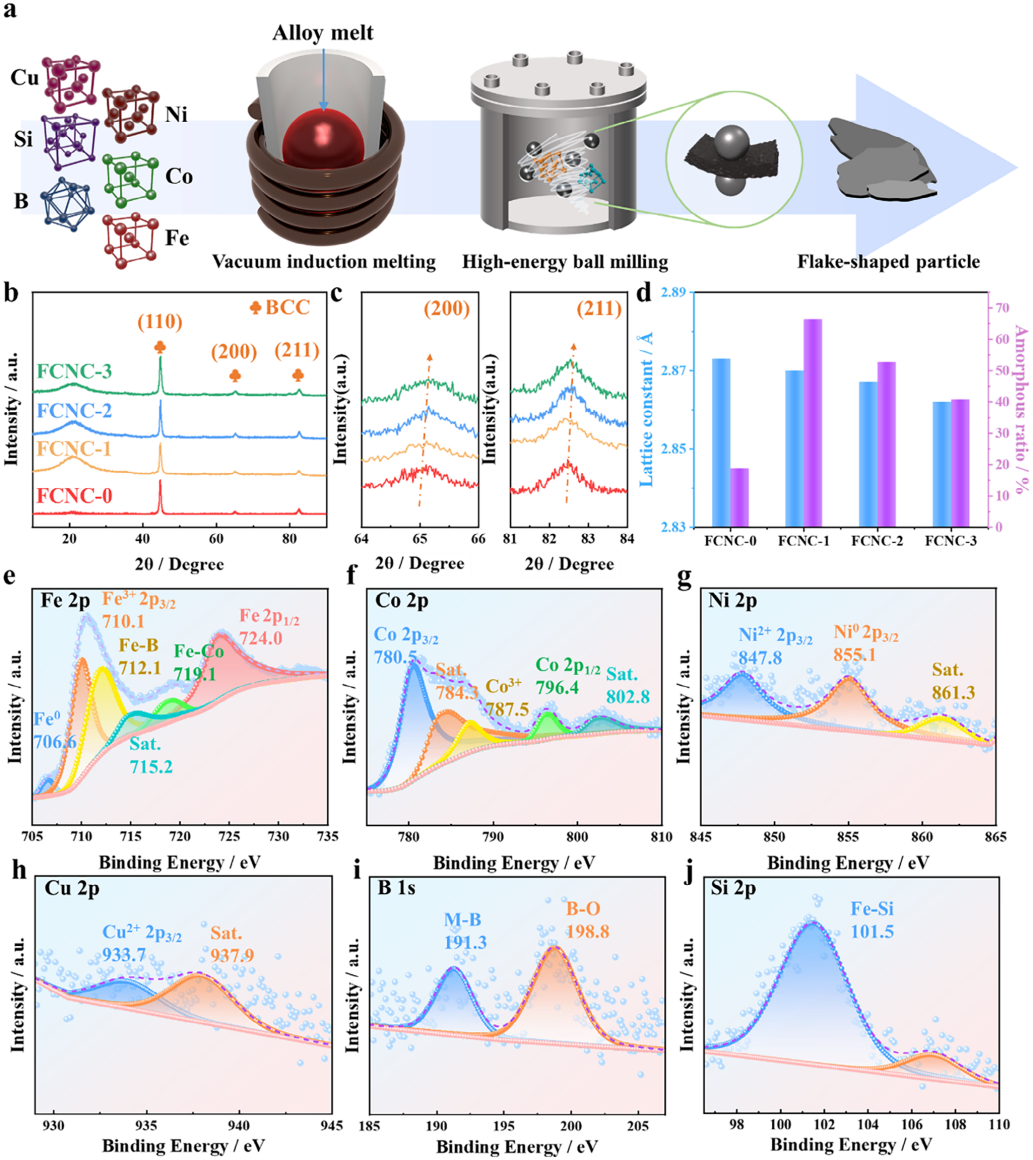
(a) Preparation flow chart; (b) XRD pattern; (c) Local magnification; (d) Lattice constant variation; (e) Fe 2p; (f) Co 2p; (g) Ni 2p; (h) Cu 2p; (i) B 1s; (j) Si 2p narrow peak spectra of FCNC‐2.

Flaky powder samples FCNC‐0 through FCNC‐3 were prepared via vacuum induction melting followed by mechanical ball milling. The induction melting step ensured uniform mixing of constituent metals under an argon atmosphere, thereby minimizing oxidation. Subsequent ball milling pulverized the bulk alloy into flaky particles with high aspect ratios. A schematic of the overall process is presented in Figure 1a. Phase identification of the as‐prepared soft magnetic alloys was carried out by X‐ray diffraction (XRD) analysis.

As shown in the XRD patterns (Figure 1b), all samples exhibit a body‐centered cubic (BCC) structure. The interplanar spacings were determined from high‐resolution TEM (HR‐TEM) imaging, and the corresponding lattice constants were calculated using Bragg's law Equation (1).

(1) $$d=\frac{\lambda }{2sin\theta }$$

where *λ* and *θ* represent the incident X‐ray wavelength and the diffraction angle, respectively. With increasing Cu doping, the lattice constant decreases from 2.873 Å (FCNC‐0) to 2.862 Å (FCNC‐3). This contraction is reflected in the XRD patterns (Figure 1c), where the characteristic (200) and (211) peaks systematically shift toward higher diffraction angles. The calculated lattice constants and the corresponding proportion of amorphous regions are summarized in Figure 1d. The lattice contraction is attributed to the substitution of Fe and Co by Cu in the BCC lattice. Although Cu possesses a larger atomic radius, its incorporation induces local lattice strain and distortion, leading to a reduction in the lattice parameter. Concurrently, Cu doping significantly increases the volume fraction of the amorphous phase. This suggests that Cu atoms act as heterogeneous nucleation sites within the amorphous matrix, promoting the formation of nanocrystallites and refining the grain structure. At higher Cu concentrations, the prolific formation of nanocrystals diminishes the amorphous content while concurrently giving rise to a high density of interfaces at the amorphous/nanocrystalline boundaries, a microstructure known to enhance electromagnetic loss.

The high‐resolution XPS spectra (Figure 1e–j and Figure S2) were deconvoluted to elucidate the chemical states of each element. In the Fe 2p spectrum (Figure 1e), the Fe 2p_3/2_ core level is fitted with multiple components: a peak at ∼710.1 eV is ascribed to Fe^3+^, while the signal at ∼724.0 eV corresponds to Fe 2p_1/2_ [37]. An additional peak at ∼719.1 eV is associated with metallic Fe in the Fe–Co alloy phase, confirming the coexistence of Fe in different oxidation and metallic states. The Co 2p spectrum (Figure 1f) displays characteristic peaks at ∼780.5 eV (Co 2p_3/2_), ∼787.5 eV (Co^3+^), and higher binding energy features near 796.4 and 802.8 eV, indicative of Co in varied chemical environments [38]. For Ni, the 2p_3/2_ peak centered at ∼847.8 eV is consistent with Ni^2+^ (Figure 1g) [39]. The Cu 2p spectrum (Figure 1h) shows a dominant 2p_3/2_ peak at ∼933.7 eV, characteristic of Cu^2+^ [40]. The B 1s region (Figure 1i) is resolved into two components: a peak at ∼191.3 eV, assigned to B in an M(Fe, Co, Ni)‐B environment, and another at ∼198.8 eV, related to B in the B‐O phase, reflecting boron's distinct local coordinations. Finally, the Si 2p peak at ∼101.5 eV (Figure 1j) confirms the presence of Si primarily within an Fe–Si bonding configuration [41].

**FIGURE 2:**
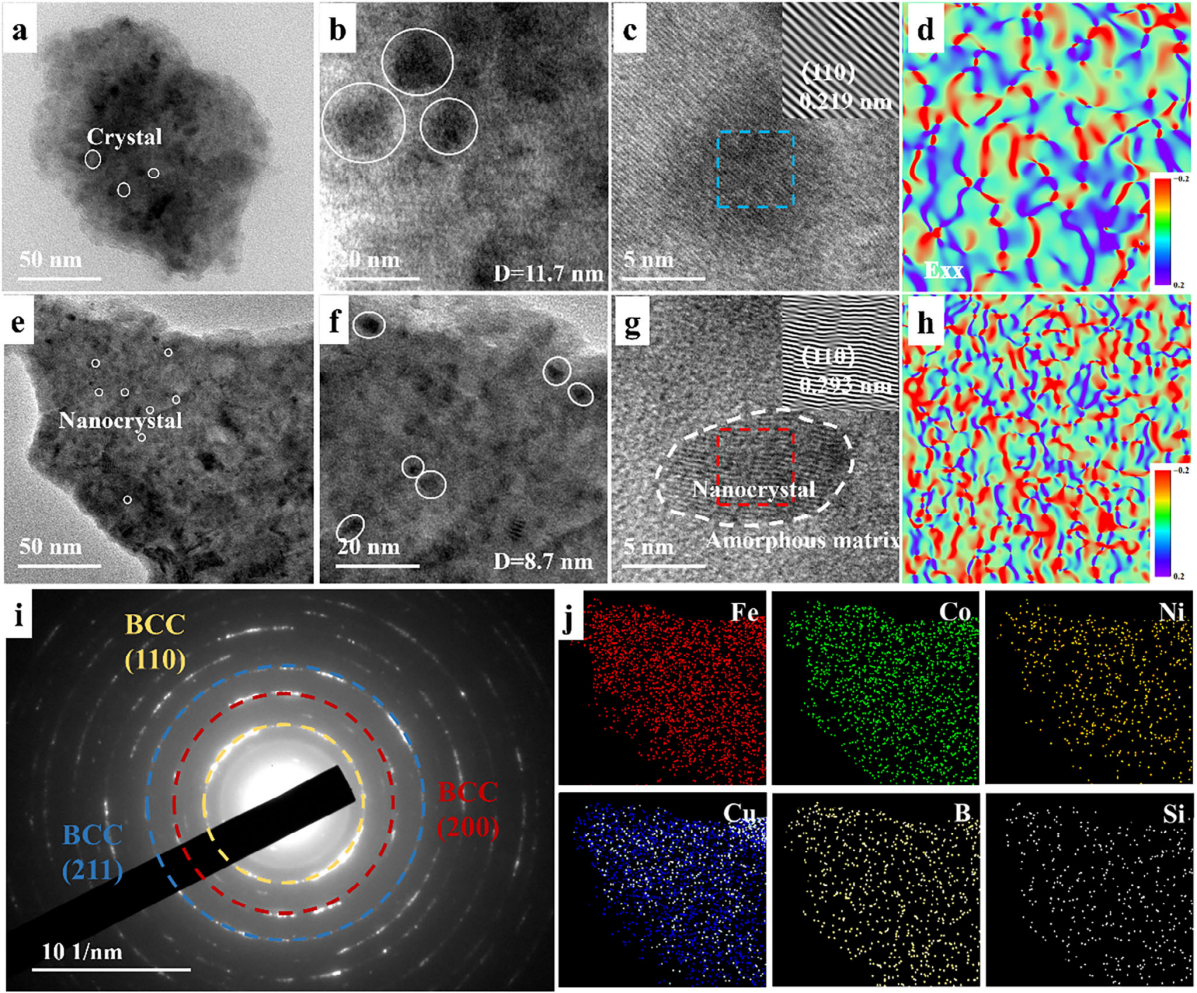
(a–d) High‐resolution TEM images, Fourier‐inverse Fourier transform results, and stress of FCNC‐0; (e–h) High‐resolution TEM images, Fourier‐inverse Fourier transform results and stress of FCNC‐2;(i) SAED pattern of FCNC‐2; (j) EDS results of FCNC‐2.

The microstructure and lattice evolution of the samples were examined by TEM. Figure 2a,e, and Figure S3 display the flaky morphology of FCNC‐0 and FCNC‐2, respectively, with FCNC‐2 exhibiting a markedly larger particle size. Both samples show evidence of crystallite precipitation. A high density of nanocrystals embedded within an amorphous matrix is evident in FCNC‐2 (Figure 2e). High‐resolution TEM (HR‐TEM) images (Figure 2b,f) reveal clear interfaces between the nanocrystals and the amorphous phase. In the region highlighted by the red box in Figure 2g, multiple stacked nanocrystals are observed. This refined structure, with crystal sizes reduced to 7–10 nm, results from the increased Cu doping, which provides additional nucleation sites.

Magnified views of local regions (Figure 2c,g) show distinct lattice fringes. In Figure 2d, fringes corresponding to the (110) plane with a spacing of 0.219 nm are identified. In contrast, Figure 2g shows significant lattice distortion and structural defects, where the interplanar spacing expands to 0.293 nm. Fourier and inverse Fourier transforms of these two regions (Figure 2d,h) were performed for further analysis. The transformation from straight to distinctly curved lattice fringes in the grain‐aggregation zone confirms the occurrence of lattice distortion. Subsequent strain mapping of the selected area reveals a transition from sparse to concentrated strain distribution, directly illustrating the lattice distortion and strain induced by Cu doping. The schematic diagram is shown in Figure S1.

The corresponding selected‐area electron diffraction (SAED) pattern (Figure 2i) exhibits a superposition of sharp diffraction spots and diffuse rings, indicating the presence of numerous randomly oriented nanocrystals. The diffraction rings can be indexed to the (110), (200), and (211) planes of a BCC structure, consistent with the XRD results. Energy‐dispersive X‑ray spectroscopy (EDS) mapping of the region in Figure 2e,j confirms a homogeneous spatial distribution of Fe, Co, Ni, and Cu, with no apparent elemental segregation. Consequently, the observed bending of lattice fringes in the TEM images originates in lattice strain or structural distortion.

## 3 Excellent Magnetic Properties

**FIGURE 3:**
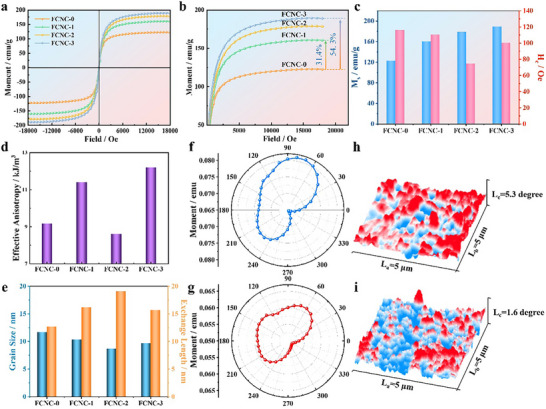
(a and b) VSM results and their magnified views; (c) *M*_s_ and *H*_c_; (d) *K*_eff_ of the samples; (e) Comparison between grain size and *L*_ex_; (f and g) Magnetocrystalline anisotropy of FCNC‐0 and FCNC‐2; (h and i) Magnetic force microscopy (MFM) images.

The soft magnetic properties of the alloys were evaluated by vibrating sample magnetometry (VSM) under an applied field of 5000 Oe (Figure 3a–c and Figure S4). All samples exhibit narrow hysteresis loops, characteristic of soft magnetic behavior. Both saturation magnetization (*M*_s_) and coercivity (*H*_c_) vary systematically with Cu doping. The *M*_s_ value increases from 122.7 emu g^−1^ for FCNC‐0 to 160.7 emu g^−1^ for FCNC‐1(a rise of 31.4%), and further rises to 189.4 emu g^−1^ for FCNC‐3, corresponding to a total enhancement of 54.3%. In contrast, *H*_c_ decreases significantly, from 116.1 Oe (FCNC‐0) to 74.8 Oe (FCNC‐2).

The enhanced soft magnetic properties are ascribed to the formation of Cu‐induced nanocrystals. Increased Cu content promotes grain refinement, and the magnetic resonance between neighboring nanocrystals further improves the magnetic softness. Grain size is critically linked to permeability: when the nanocrystal dimensions fall below the single‐domain threshold, the magnetocrystalline anisotropy increases, shifting the resonance frequency (*f*_ex_) toward higher values [22]. The resonance frequency can be expressed as:

(2) $$f_{ex}\;\propto \frac{A}{D^{2}}$$

(3) $$H_{k}=\frac{2K}{\mu_{0}M_{s}}$$

(4) $$H_{k}=\frac{H_{c}}{\alpha }$$

where *µ*_0_ is the vacuum permeability (4π × 10^−7^ H m^−1^) and *α* is an empirical correction factor (typically ranging from 0.3 to 0.6 for polycrystalline materials). Based on Equations ((2), (3), (4)), the increase in *M*_s_ and decrease in *H*_c_ with Cu doping predict a shift in the material's natural resonance frequency toward higher values.

For nanocrystalline systems, the resonance frequency is closely related to the *L*_ex_ [22], which is expressed as:

(5) $$L_{ex}=\sqrt{\frac{A}{K_{eff}}}$$

where *A* denotes the exchange stiffness, which characterizes the strength of the exchange interaction, and *K*_eff_ represents the effective anisotropy constant, which incorporates contributions from magnetocrystalline, shape, and stress anisotropies [42]. The *K*_eff_ derived from the coercivity formula is presented in Figure 3d.

(6) $$K_{eff}=\frac{H_{c}\mu_{0M_{s}}}{P_{c}}$$

with *p*_c_ ≈ 0.2, the *K*_eff_ calculated from the *M*_s_ and *H*_c_ values (obtained by VSM) are 9.2 kJ m^−13^ for FCNC‐0 and 8.6 kJ m^−13^ for FCNC‐2. This result confirms that Cu doping effectively reduces the magnetocrystalline anisotropy of the samples, leading to a lower *K*_eff_. As illustrated in Figure 3e and Figure S5, the calculated *L_ex_* is compared with the experimentally measured average *D*. The values of *D* are 11.7 nm for FCNC‐0 and 8.7 nm for FCNC‐2, both significantly smaller than the corresponding *L_ex_* of about 19.1 nm. This size relationship (*D* < *L_ex_*) is characteristic of soft magnetic systems and directly accounts for the pronounced decrease in coercivity. Collectively, the VSM measurements and *L_ex_* analysis demonstrate that Cu doping refines the grain structure to dimensions well below the exchange length. This refinement enhances intergranular exchange coupling and optimizes the soft magnetic properties, which is a crucial factor underlying the improved high‐frequency electromagnetic absorption performance.

The magnetocrystalline anisotropy of FCNC‐0 and FCNC‐2 was characterized, as shown in Figure 3f,g. Both samples exhibit maximum saturation magnetization near an orientation of approximately 70°. On the same scale, FCNC‐0 displays more pronounced magnetic anisotropy. This difference arises because Cu doping lowers the nucleation barrier, refines the nanocrystal size, and allows more grains to be encompassed within the *L*_ex_. Consequently, the magnetic domains of adjacent grains tend to align parallel to one another, leading to an overall reduction in magnetic anisotropy.

To better resolve magnetic domains and domain walls, magnetic force microscopy (MFM) was employed. The effect of Cu‐induced nanocrystallization on the magnetic domain structure was investigated by comparing the static domain configurations of FCNC‐0 and FCNC‐2. Powder samples were embedded in an epoxy resin with a resin‐to‐curing agent mass ratio of 2:1 and subsequently polished to ensure a flat surface for MFM measurements. As shown in Figure 3h,i, the bright contrast corresponds to domain walls. Compared with the sparsely distributed domain walls in FCNC‐0, FCNC‐2 exhibits a significantly higher density of domain walls and pronounced domain fluctuations, which are conducive to enhancing domain wall resonance loss. According to the expression for domain wall width: [43]

(7) $$\sigma =\pi \;\sqrt{\frac{A}{K}}$$

Following Cu doping, the grain size is refined to 8.7 nm, while the magnetic domain size expands from 39.8 nm to 60.0 nm. The exchange stiffness (shown in Figure S6), calculated via Equation (7), increases from 1.5 × 10^−12^ to 3.1 × 10^−12^ J m^−1^, favoring the optimization of *L*_ex_. Since domain wall motion must overcome an energy barrier, a high domain wall density results in a shorter travel distance and lower energy expenditure per wall. Consequently, a high domain wall density typically corresponds to low coercivity, consistent with the reduced *H*_c_ observed for FCNC‐2 in the VSM measurements. The initial permeability (*µ*_i_) can be related to *L*_ex_ as follows: [44]

(8) $$\mu_{i}\;\propto \frac{\mu_{0M_{s}^{2}}}{K_{eff}}\;\propto \frac{M_{s}^{2}L_{ex}^{2}}{A}$$

A higher density of domain walls implies a greater number of mobile walls, as magnetization can be achieved through the small‐scale displacement of a larger population of walls. This mechanism is expected to enhance the initial permeability, suggesting that FCNC‐2 likely possesses a superior *µ*_i_ value.

Based on the definition of the magnetic exchange length *L*_ex_, its magnitude is influenced by the competition between exchange energy and magnetic anisotropy. Fe and Co, being ferromagnetic elements, contain unpaired electrons in their 3d orbitals. The introduction of metalloid elements facilitates effective electron hybridization with these unpaired electrons, thereby enhancing the local magnetic moments in Fe–Co‐based soft magnetic alloys. Within an appropriate composition range, *L*_ex_ can thus be tuned by varying the ratio of ferromagnetic to metalloid elements. Furthermore, when nucleation‐promoting elements such as Cu are doped into the Fe–Co alloy, they effectively lower the nucleation barrier and promote nanocrystallization, resulting in a significant reduction in grain size.

The formation of nanocrystals introduces internal stress and shortens the interatomic electron‐cloud distances. The resulting overlap of electron clouds restricts magnetization along certain directions and hinders the motion of internal magnetic moments. These effects collectively lead to a significant reduction in coercivity, an indirect increase in saturation magnetization, a decrease in the effective magnetic anisotropy constant, and ultimately, the enhancement of magnetic exchange interactions [22].

## 4 Flaky Wave Absorbing Device

**FIGURE 4:**
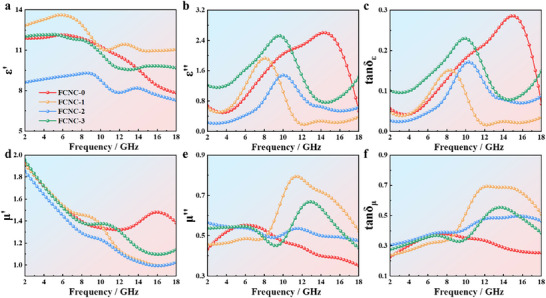
(a) Real part of permittivity; (b) Imaginary part of permittivity; (c) Dielectric loss factor; (d) Real part of permeability; (e) Imaginary part of permeability; (f) Magnetic loss factor of FCNC samples.

Figure 4 presents the electromagnetic parameters of the samples. The microwave absorption characteristics are primarily governed by the complex permittivity (*ε*_r_) and complex permeability (*µ*_r_). The real parts (*ε′* and *µ′*) reflect the material's ability to store electrical and magnetic energy, whereas the imaginary parts (*ε″* and *µ″*) characterize its capability to attenuate electromagnetic energy.

Figure 4a reveals a substantial variation in *ε′* with Cu doping, reaching a maximum for FCNC‐1. This can be attributed to the synergistic effect of Cu, which promotes nanocrystal formation, and Ni, which modulates atomic diffusion, thereby controlling the material's microstructure. Notably, at higher Ni and Cu contents, the *ε′* curves exhibit similar trends, suggesting that these two elements play complementary roles in tailoring nanocrystal size and distribution.

Analysis of the *ε″* in Figure 4b shows that FCNC‐1, FCNC‐2, and FCNC‐3 exhibit multiple relaxation peaks. This behavior can be explained by Debye relaxation theory, indicating the presence of multiple polarization relaxation processes within this frequency band—a key feature contributing to the samples' broadband absorption capability. Based on the characteristic frequencies of the relaxation peaks observed in the dielectric spectrum, the polarization relaxation below 10.0 GHz can be primarily attributed to defect‑dipole effects arising from point defects or impurities within the crystalline lattice. In contrast, the relaxation peaks above 10.0 GHz mainly originate from lattice‑distortion‑induced polarization, associated with the localized strain fields inside the nanocrystals.

Figure 4d,e shows that the samples exhibit multi‐peak magnetic responses above 10.0 GHz. The FCNC‐1 and FCNC‐3 samples maintain relatively high magnetic loss (*µ″* ≥ 0.4) across the entire band, whereas FCNC‐0 and FCNC‐2 display distinct polarization peaks near 16.0 GHz, which are associated with spin‐resonance behavior of magnetic dipoles under an alternating electromagnetic field.

A comparison of the Cu‑doping effects shows that µ″ initially increases and then decreases as Ni is progressively replaced by Cu, with FCNC‑2 exhibiting the highest loss in the 10.0–16.0 GHz range. To further distinguish the underlying loss mechanisms, the magnetic loss tangent (*tanδ_µ_* = *µ″*/*µ′*) was analyzed and deconvoluted (Figure 4f and Figure S7). The deconvolution confirms that the enhanced *µ′′* of FCNC‑2 arises from two superimposed contributions: a broad, low‑frequency background attributed to domain‑wall motion and a sharp, high‑frequency peak centered near 12 GHz, which is characteristic of exchange‑driven resonance. This resonance originates from the strong intergranular exchange coupling established when the grain size becomes much smaller than the *L_ex_*. Combined with XRD analysis, the incorporation of smaller Cu atoms into the lattice induces contraction and distortion, generating point defects. This behavior is attributed to the synergistic change in magnetic defect concentration and the arrangement of atomic magnetic moments.

**FIGURE 5:**
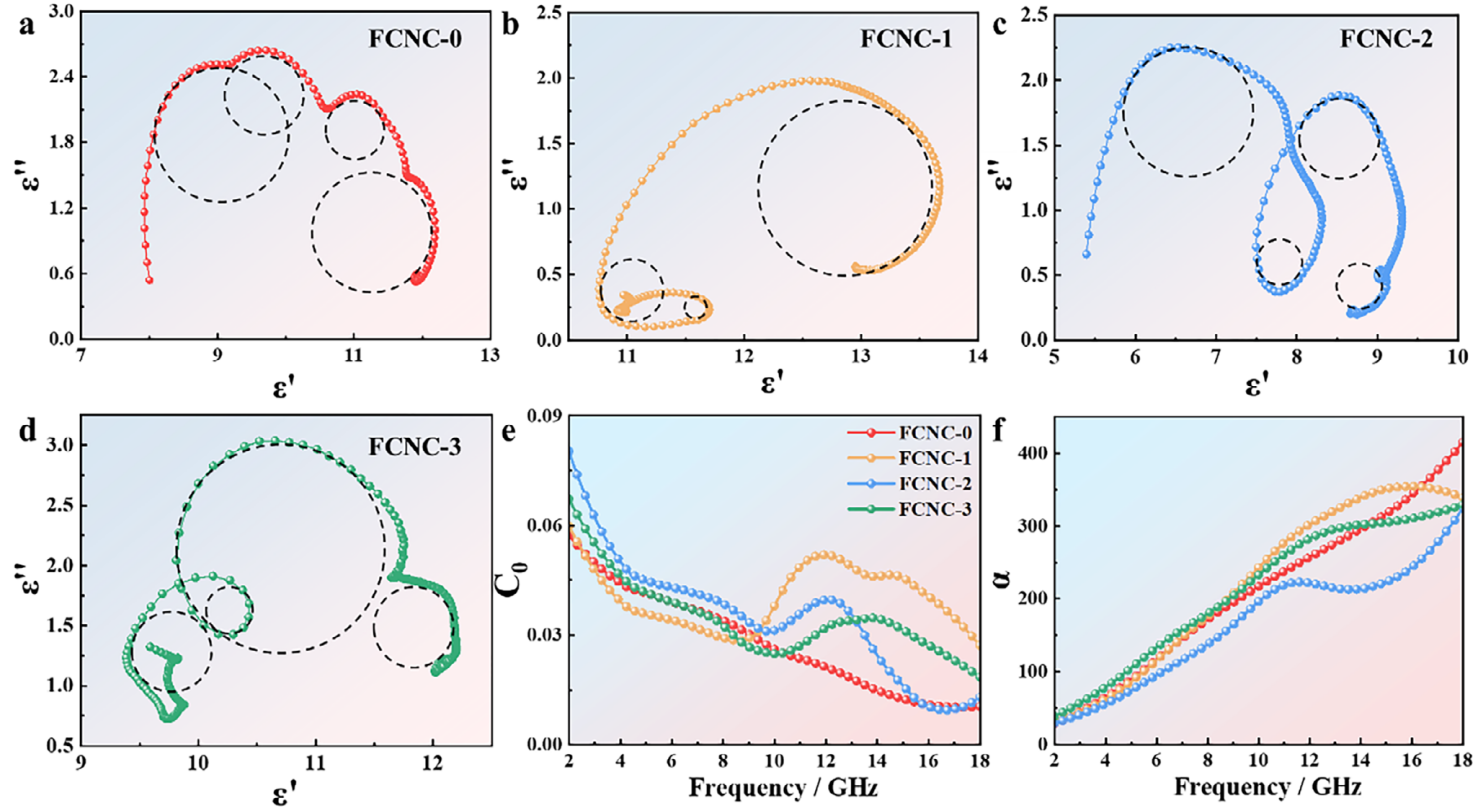
(a–d) Cole‐Cole plots of FCNC‐0, FCNC‐1, FCNC‐2, and FCNC‐3; (e) value of eddy current loss (*C*_0_); (f) Attenuation constants.

Electromagnetic wave attenuation arises from both dielectric loss and magnetic loss, corresponding to variations in complex permittivity and permeability, respectively. Given that the permittivity changes more prominently with composition, dielectric loss is inferred to be the dominant attenuation mechanism in these samples. Within the GHz range, the observed dielectric loss originates primarily from dipole and interfacial polarization. In Debye theory, polarization relaxation processes are often represented by Cole–Cole semicircles, where each semicircle corresponds to a distinct polarization mechanism. The Cole–Cole plots of the samples are presented in Figure 5 to elucidate the influence of polarization relaxation on the reflection loss.

The Cole–Cole plots of the four samples are presented in Figure 5a–d. The presence of multiple, distinct semicircles indicates several dielectric relaxation processes within the materials. This behavior is attributed to the abundance of grain boundaries, featuring an amorphous atomic structure coupled with a high defect density and localized stress fields. As Cu doping increases, the number of discernible semicircles grows, reflecting the microstructural evolution induced by Cu. The substitution of Fe by Cu causes lattice mismatch and distortion, promoting the migration and accumulation of localized electrons. Under an alternating electric field, the accumulation and redistribution of charges at grain boundaries lead to interfacial polarization. The finite time required for this charge redistribution results in grain‑boundary relaxation.

Magnetic loss in magnetic materials is governed by the *µ″* and can stem from hysteresis loss, domain‑wall resonance, eddy‑current loss, natural resonance, and exchange resonance. Hysteresis loss is negligible in weak fields, and domain‑wall resonance typically occurs in the MHz range and can be disregarded here. If magnetic loss were solely due to eddy currents, the coefficient *C*_0_ (calculated using Equation 9) [45] would remain constant with frequency. Otherwise, the loss is dominated by exchange resonance.

(9) $$C_{0}=\mu^{\prime \prime }{\left(\mu^{\prime }\right)}^{-2}f^{-1}$$

Figure 5e shows a distinct peak at 12.0 GHz in all Cu‐doped samples, which corresponds to exchange resonance. The intensity of this resonance peak gradually diminishes with increasing Cu (and decreasing Ni) content. This trend may be attributed to the rise in material permeability. Following the principles of electromagnetic induction, increased permeability is directly linked to amplified magnetic flux variation, leading to a stronger induced electromotive force and, consequently, elevated eddy‐current loss. Excessively high permeability can also cause rapid motion of magnetic domains and accelerated energy dissipation, thereby suppressing the resonance intensity.

Based on the measured electromagnetic parameters, the attenuation constant (*α*) of the materials was calculated using the following formula: [46, 47]

(10)

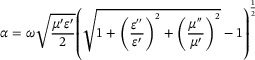




The attenuation constants (*α*) for all four samples are plotted in Figure 5f. Each exhibits an increasing trend from 2.0 to 18.0 GHz, indicating that higher frequencies enhance the magnetic‐loss‐related attenuation. The curve for FCNC‐0 is relatively flat and featureless. In contrast, FCNC‐1, with a low Cu content, manifests enhanced frequency dependence at elevated frequencies, suggesting that its internal magnetic‐domain flipping and eddy‐current loss are strongly frequency‐dependent. With further Cu doping, FCNC‐2 displays the lowest attenuation among the four. At this composition, the arrangement of magnetic moments and the exchange interaction within the material undergo a frequency‐dependent “loss redistribution,” which is favorable for achieving a broader effective absorption bandwidth (EAB) at high frequencies.

**FIGURE 6:**
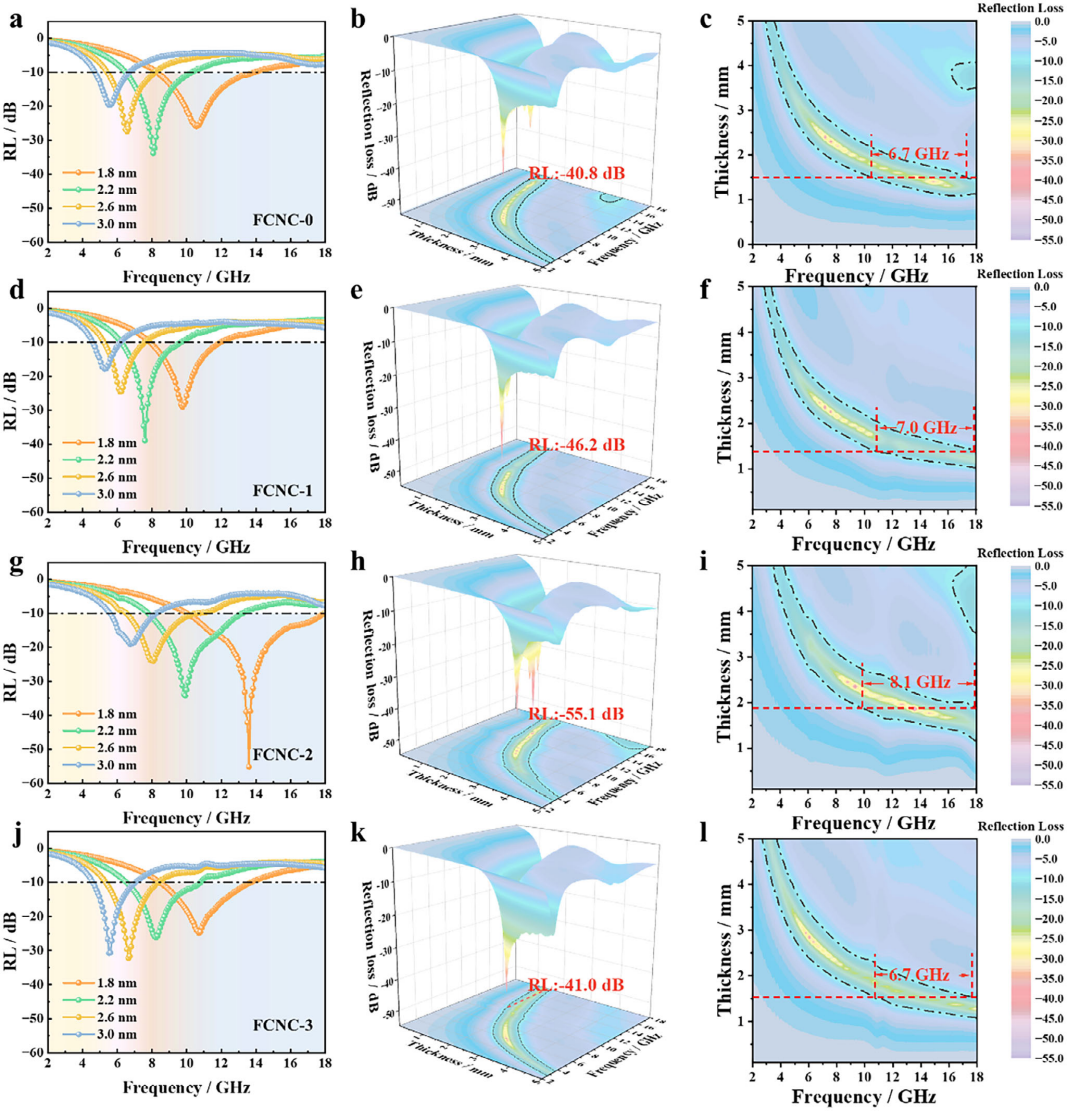
(a–c) FCNC‐0; (d–f) FCNC‐1; (g–i) FCNC‐2; (j–l) 2D and 3D reflection loss maps of FCNC‐3.

Reflection Loss (RL) is a key parameter that quantifies the energy ratio between reflected and incident waves at an interface, arising from the mismatch in electromagnetic properties (e.g., permittivity and permeability). Based on the measured electromagnetic parameters, RL can be calculated. For a single‐layer absorber, the normalized input impedance can be derived from the complex permittivity and permeability using Equation (11): [48]

(11) $$Z_{in}=Z_{0}\sqrt{\frac{\mu_{r}}{\varepsilon_{r}}}\tanh\left(j\frac{2\pi fd}{c}\sqrt{\mu_{r}\varepsilon_{r}}\right)$$

where *µ*_r_ and *ε*_r_ are the complex permeability and permittivity, respectively; *Z*_0_ is the impedance of free space; *c* is the speed of light; *f* is the frequency; and *d* is the thickness of the absorber. The RL can then be obtained by Equation (12) [49]:

(12) $$RL=20log\left|\;\left(Z_{in}-Z_{0}\right)/\left(Z_{in}+Z_{0}\right)\right|$$

based on the measured electromagnetic parameters, the RL was calculated. The resulting frequency‐dependent RL curves are presented in Figure 6.

The EAB (defined as the frequency range where RL < −10 dB) and the minimum reflection loss (RL_min_) were evaluated based on the electromagnetic parameters. As shown in Figure 6a–c, FCNC‐0 achieves an RL_min_ of −40.8 dB at 17.2 GHz with a thickness of 1.2 mm, and an EAB of 6.7 GHz (10.6–17.3 GHz) at 1.5 mm. With initial Cu doping, FCNC‐1 (Figure 6d–f) exhibits an improved RL_min_ of −46.2 dB at 7.2 GHz (2.3 mm) and a broader EAB of 7.0 GHz (11.0–18.0 GHz). When the Cu content is further increased, the FCNC‐2 sample delivers the optimum microwave absorption performance among all studied compositions. As shown in Figure 6g–i, this sample achieves a RL_min_ of −55.1 dB at 13.6 GHz with a thickness of only 1.8 mm, and exhibits an EAB of 8.1 GHz (covering 9.3–17.4 GHz) at the same thickness. These results confirm that the enhancement in absorption originates from the synergistic effects of Cu doping: promoting nanocrystallization and inducing lattice distortion, which collectively optimize the impedance matching and attenuation characteristics.

The partial substitution of Fe by Cu introduces lattice mismatch stress due to the atomic size difference, which lowers the phase transformation barrier and facilitates nanocrystal precipitation. The resulting nanocrystals help tailor the exchange resonance frequency. Simultaneously, the Cu‐induced lattice distortion creates local strain fields, while associated vacancies and grain boundaries enhance interfacial polarization and dipole relaxation. Consequently, the impedance matching is optimized (as shown in Figure S8), leading to a significantly broadened effective absorption bandwidth, which is enhanced by 47.1% at a thickness of 1.8 mm. However, excessive Cu doping (FCNC‐3) leads to deteriorated RL_min_ and EAB. This decline is attributed to the over‑reduced nucleation barrier, which triggers grain coalescence and coarsening. Consequently, the *H*_c_, which reaches a minimum in FCNC‐2, increases again in FCNC‐3, lowering the exchange resonance frequency and weakening the magnetic softness—factors that ultimately impair the absorption performance.

**FIGURE 7:**
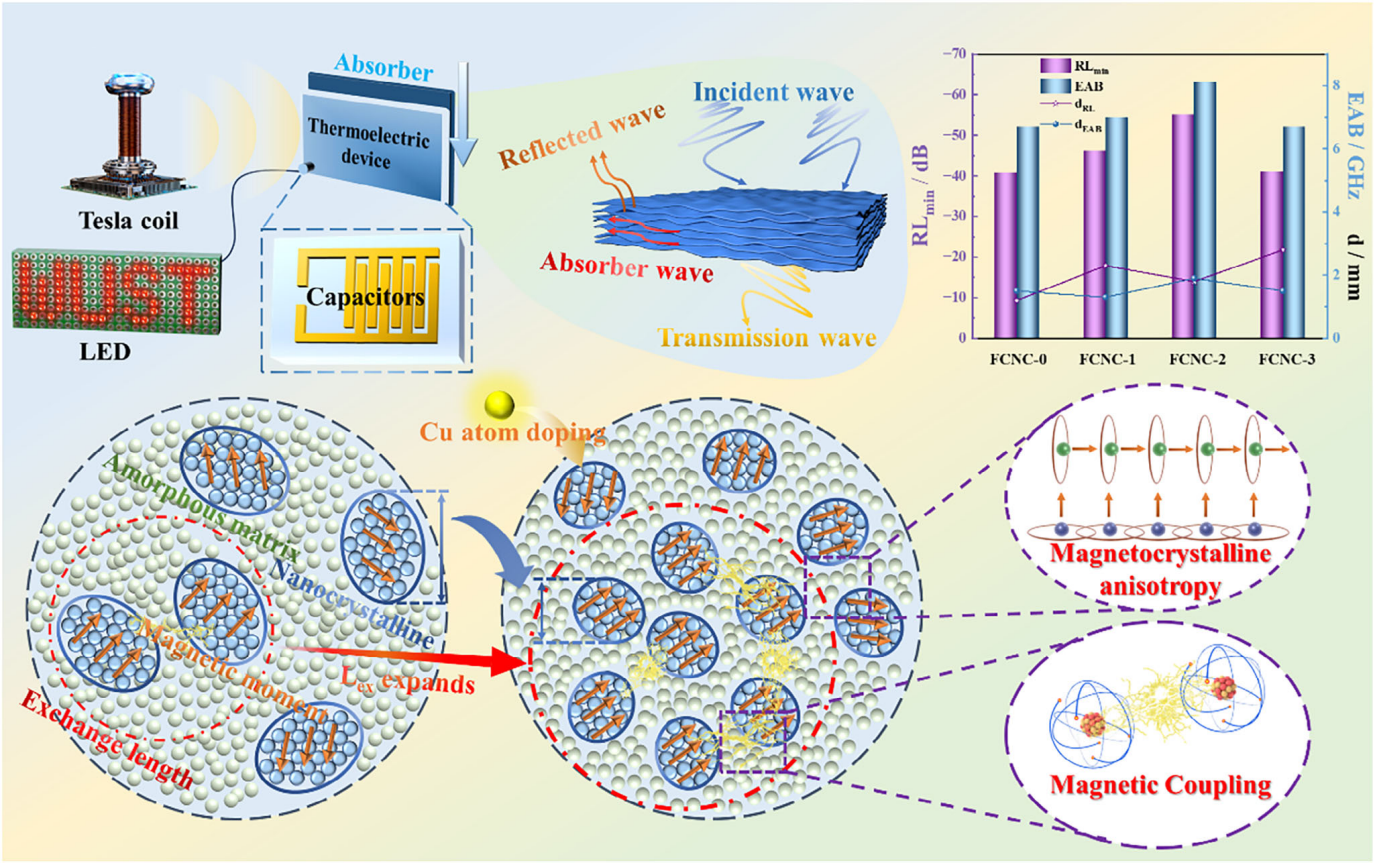
(a) Comparison of EAB under different thicknesses; (b) RL and EAB of all samples in this work; (c) The principle diagram of magneto‐optical converter based on flexible absorbing materials; (d) LED brightness before and after using the absorber; (e) Mechanism diagram of *L*_ex_‐guided intergranular coupling.

Figure 7a presents a comparative scatter plot of the microwave absorption performance for various Fe‐based amorphous soft magnetic materials, highlighting the excellent performance of FCNC‐2 at relatively low matching thicknesses. The histogram in Figure 7b further demonstrates that Cu doping effectively optimizes the absorption characteristics of the FeCoSi‐based soft magnetic composite. Leveraging a synergistic loss effect from four primary attenuation mechanisms, the FCNC‐2 sample achieves a minimum reflection loss of −55.1 dB and an effective absorption bandwidth of 8.1 GHz (RL ≤ −10 dB) at a thickness of 1.8 mm and a frequency of 13.6 GHz.

Devices for wave absorption and thermoelectric conversion fabricated using FCNC‐2 are illustrated in Figure 7c,d, respectively. When the absorber is placed between thermocouples, it absorbs electromagnetic waves emitted from a Faraday coil, converting electrical energy into thermal energy. This thermal energy is subsequently reconverted into electricity by the thermocouples, lighting a bulb from an initially off state. The schematic illustrates that approximately 90% of the incident wave energy is absorbed, with the remainder being reflected or transmitted.

Figure 7e depicts the microstructural evolution associated with grain refinement in the present work. Within the *L*_ex_, the intrinsic magnetic moments of adjacent grains are forced to align parallel under strong exchange interactions, thereby reducing the macroscopic magnetic anisotropy. Because the grain size is significantly smaller than *L*_ex_, exchange coupling effectively penetrates grain boundaries and collectively couples numerous fine grains. This coupling suppresses the inherent magnetic anisotropy of individual grains, forcing the magnetic moments to abandon their random easy‐axis orientations and adopt a nearly uniform alignment. Furthermore, the amorphous matrix between grains exhibits ferromagnetic behavior, resulting in robust intergranular magnetic coupling. This mechanism effectively enhances exchange resonance and broadens the electromagnetic wave absorption bandwidth.

## 5 Conclusions

This work presents a *L*_ex_‐guided strategy for microstructural engineering in soft magnetic alloys. Trace Cu doping in the FeCo‐based system promotes the formation of a nanocomposite structure comprising nanocrystals (∼8.7 nm) embedded in an amorphous matrix. Crucially, the grain size is significantly smaller than the measured exchange length (*L*_ex_ ≈ 19.1 nm). This configuration enables strong intergranular exchange coupling, which collectively aligns magnetic moments across grain boundaries. This effect reduces the *K*_eff_ from 9.2 to 8.6 kJ m^−13^ and shifts the exchange resonance frequency to 12.0 GHz. Furthermore, it synergistically enhances dielectric loss (via interfacial polarization and lattice distortion) and magnetic loss (via exchange and domain‐wall resonance). As a result, the optimized alloy achieves a broad EAB of 8.1 GHz at a minimal thickness of 1.8 mm, representing a 47.1% performance improvement. This study confirms that manipulating magnetic coupling through *L*_ex_ engineering is an effective strategy to mitigate high‐frequency magnetic loss in soft magnetic alloys, offering a promising pathway for developing advanced soft magnetic absorbers.

## 6 Experimental Section Methods

Raw materials used in the experiment include irregular iron sheets (Fe, 99.97%), cobalt blocks (Co, 99.5%), nickel blocks (Ni, 99.99%), high‐purity copper (Cu, 99.9999%), high‐purity boron (B, 99.99%), and high‐purity silicon (Si, 99.9999%). All raw materials were purchased online and used directly without further purification.

The above raw materials were alloyed by vacuum induction melting under an argon atmosphere to prepare (Fe_0.8_Co_0.2_)_85_B_11_Si_2_Ni_2‐x_Cu_x_ (x = 0, 0.7, 1.3, 1.5) alloy ingots, named FCNC‐0, FCNC‐1, FCNC‐2, and FCNC‐3, respectively. The crushed alloy blocks were milled using a QM‐3SP2 planetary ball mill (Nanjing University Instrument Co., Ltd.). The milling medium was WC balls with a mass ratio of 15 mm:10 mm:6 mm = 1:2:2. The total mass ratio of milling medium, anhydrous ethanol, and powder was 16:2:1. The total milling time was 30 h, with a cycle of 30 min working and 10 min stopping at 360 r min^−1^. After milling, the powder was dried in a vacuum drying oven at 50°C for 10 h, sieved through a 180‐mesh sieve, and the fine powder was collected.

The structure of the powder was analyzed using an X‐ray diffractometer (XRD, PANalytical, Bruker, Rigaku) with Cu Kα radiation, a scanning rate of 4°min^−1^, and a scanning angle of 10°∼90°. Raman spectroscopy (SR‐500 I‐A) and electron diffraction (SAED) were used to determine the crystal structure of the samples. The surface morphology of the samples was observed and analyzed using a scanning electron microscope (SEM, JSM‐7800 F). Meanwhile, the element content and distribution were analyzed by energy dispersive spectroscopy (EDS). The microstructure of the alloy was characterized using a transmission electron microscope (TEM, JEM‐2800). The powder was mixed with paraffin at a mass ratio of 6:4 in a 60°C water bath. After cooling to room temperature, the sample was pressed into a hollow ring with a thickness of 3.0 mm, an inner diameter of 3.04 mm, and an outer diameter of 7.00 mm for electromagnetic parameter testing. A vector network analyzer (NIAN, Anritus 2∼18 MS46322B) was used to test its electromagnetic parameters in the 2–18 GHz range.

## Funding

This work is supported by National Key Research and Development Program of China (2024YFB3714603), National Natural Science Foundation of China (U2541259, 52304410) and Major Project of Hubei Province (Functional Coating and Materials, 2023BAA003‐1)

## Conflicts of Interest

The authors declare no conflicts of interest.

## Supporting information




**Supporting file**: advs74207‐sup‐0001‐SuppMat.docx

## Data Availability

The data that support the findings of this study are available from the corresponding author upon reasonable request.
