# Supplementary material for: Exchange Length Tailored Magnetic Resonance for Broadband Absorption in FeCo‐Based Alloys
Source: Adv Sci (Weinh). 2026 Feb 5;13(20):e74207. doi: 10.1002/advs.74207 (PMC13067767; doi:10.1002/advs.74207)
Supplement: Supplementary file 1 — Supporting file: advs74207‐sup‐0001‐SuppMat.docx [file ADVS-13-e74207-s001.docx]

Supporting Information

**Exchange Length tailored Magnetic Resonance for Broadband Absorption in FeCo-based Alloys**

Xiaoyang Liu^a, b^, Yingli Zhu^a, b^*, Shuangxin Zhang^a, b^, Gangtao Luo^a, b^, Mengke Qiao^a, b^, Jiang Wu^a, b^, Xiangcheng Li^a, b^*

a State Key Laboratory of Advanced Refractories, Wuhan University of Science and Technology, Wuhan 430081, P. R. China

b Key Laboratory of High Temperature Electromagnetic Materials and Structure of MOE, Wuhan University of Science and Technology, Wuhan 430081, China

E-mail: [yinglizhu@wust.edu.cn](mailto:yinglizhu@wust.edu.cn), [lixiangcheng@wust.edu.cn](mailto:lixiangcheng@wust.edu.cn)


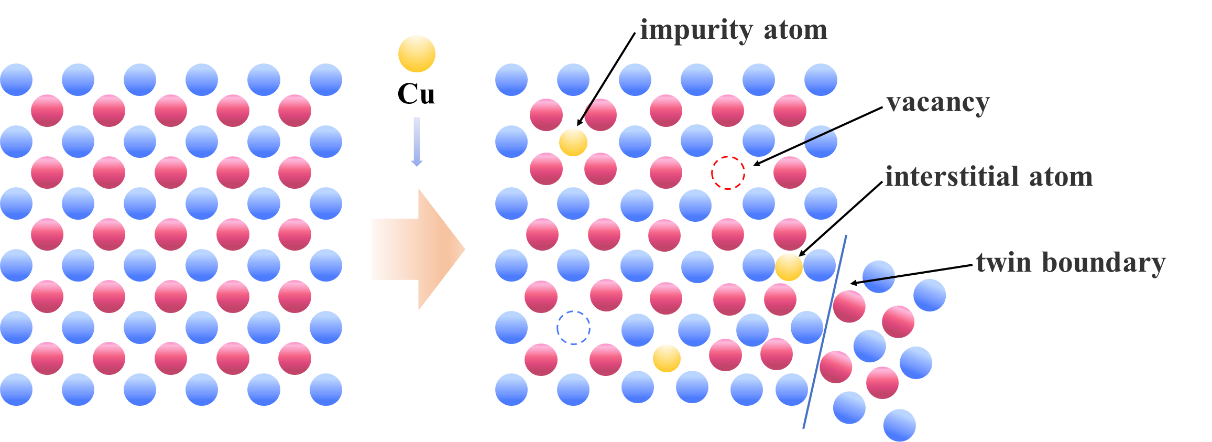


1. The indication of lattice distortion caused by Cu doping

**
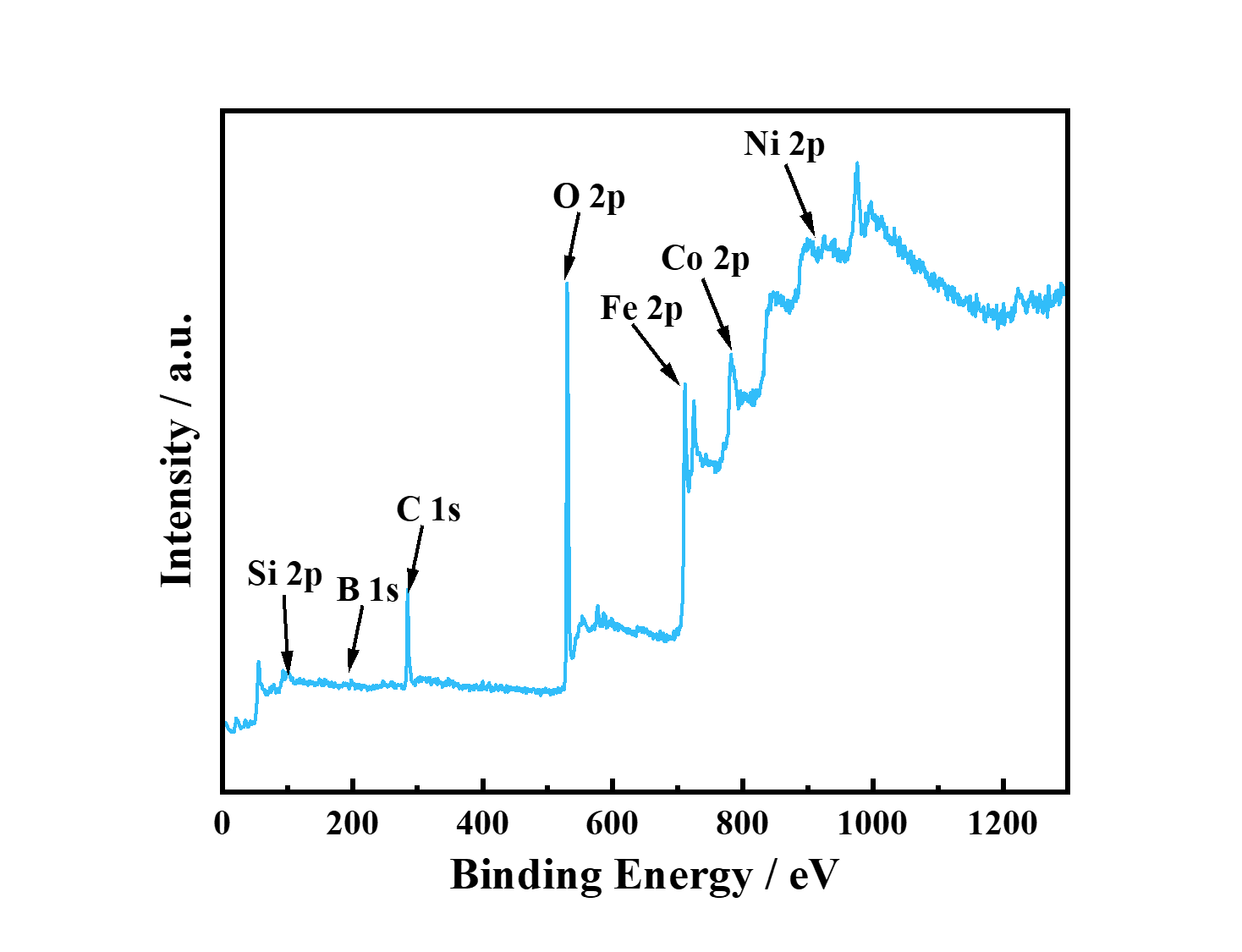
**

1. XPS of FCNC-2

**
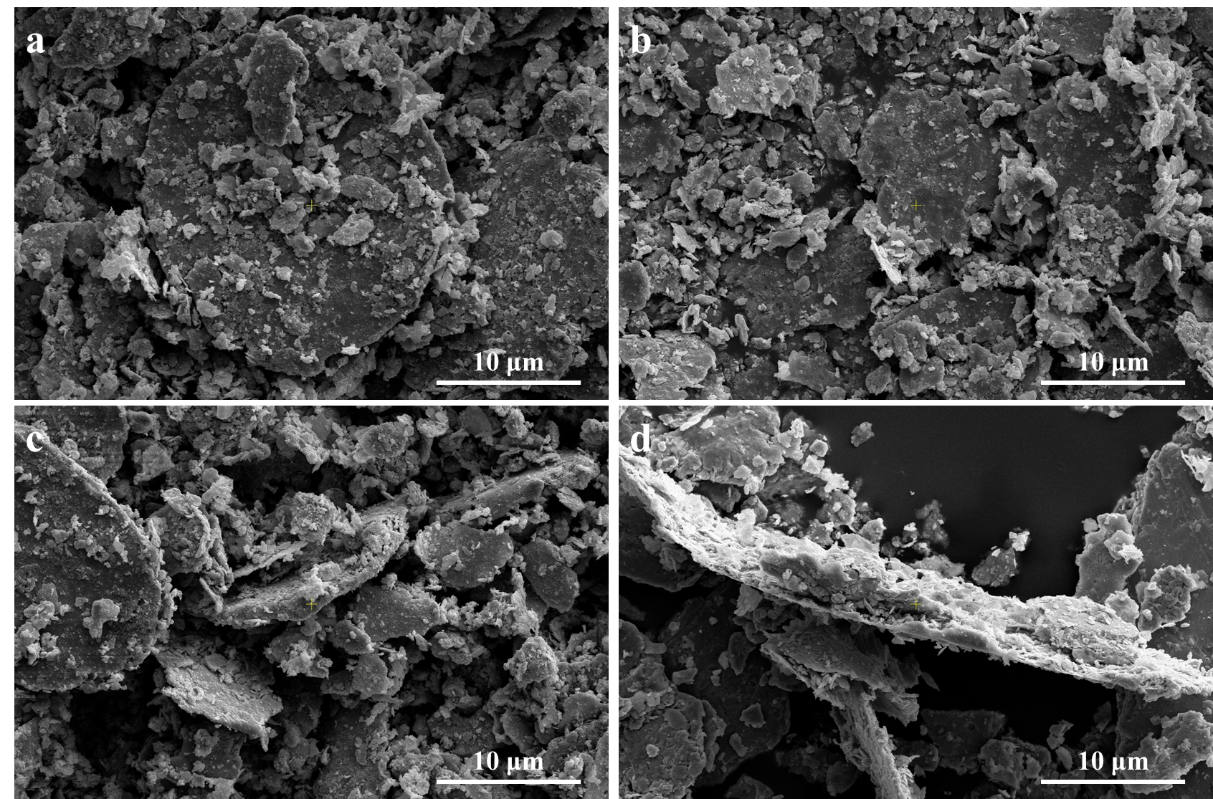
**

1. SEM images of (a) FCNC-0; (b) FCNC-1; (c) FCNC-2; (d) FCNC-3

1. *H*_c_ images of FCNC-0; FCNC-1; FCNC-2 and FCNC-3


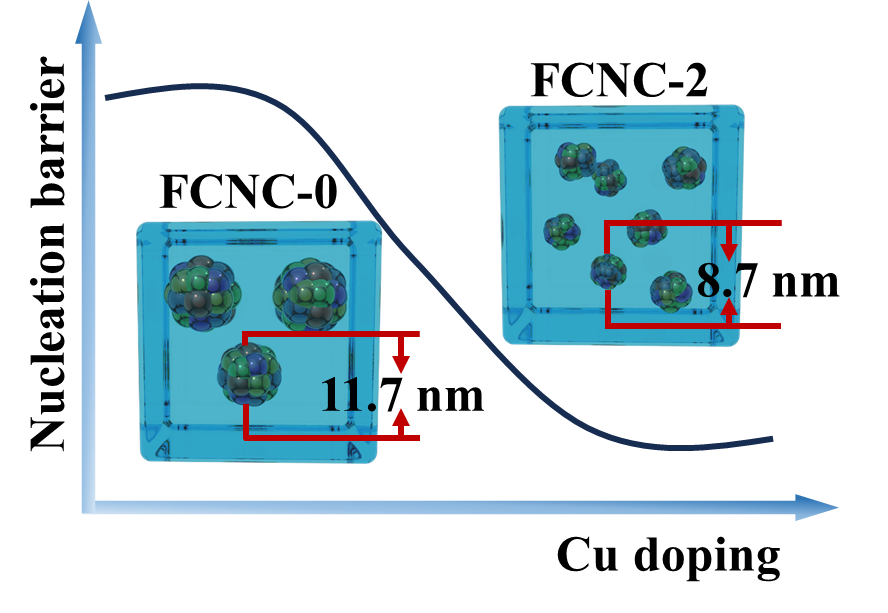


1. Cu doping refines grains

**
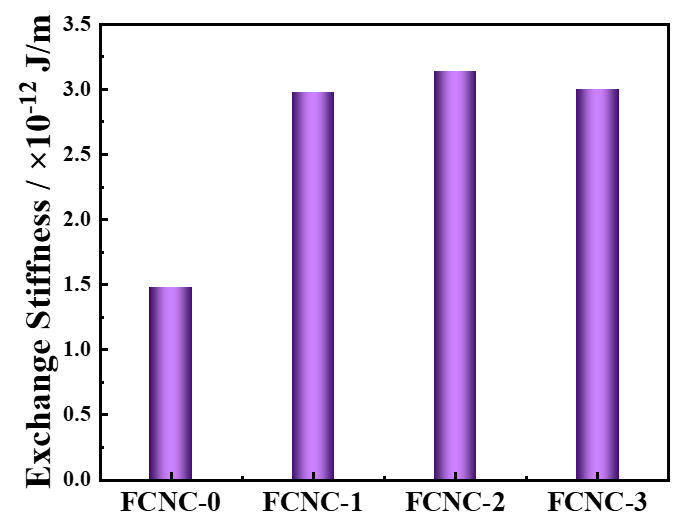
**

1. Exchange stiffness images of FCNC-0; FCNC-1; FCNC-2 and FCNC-3


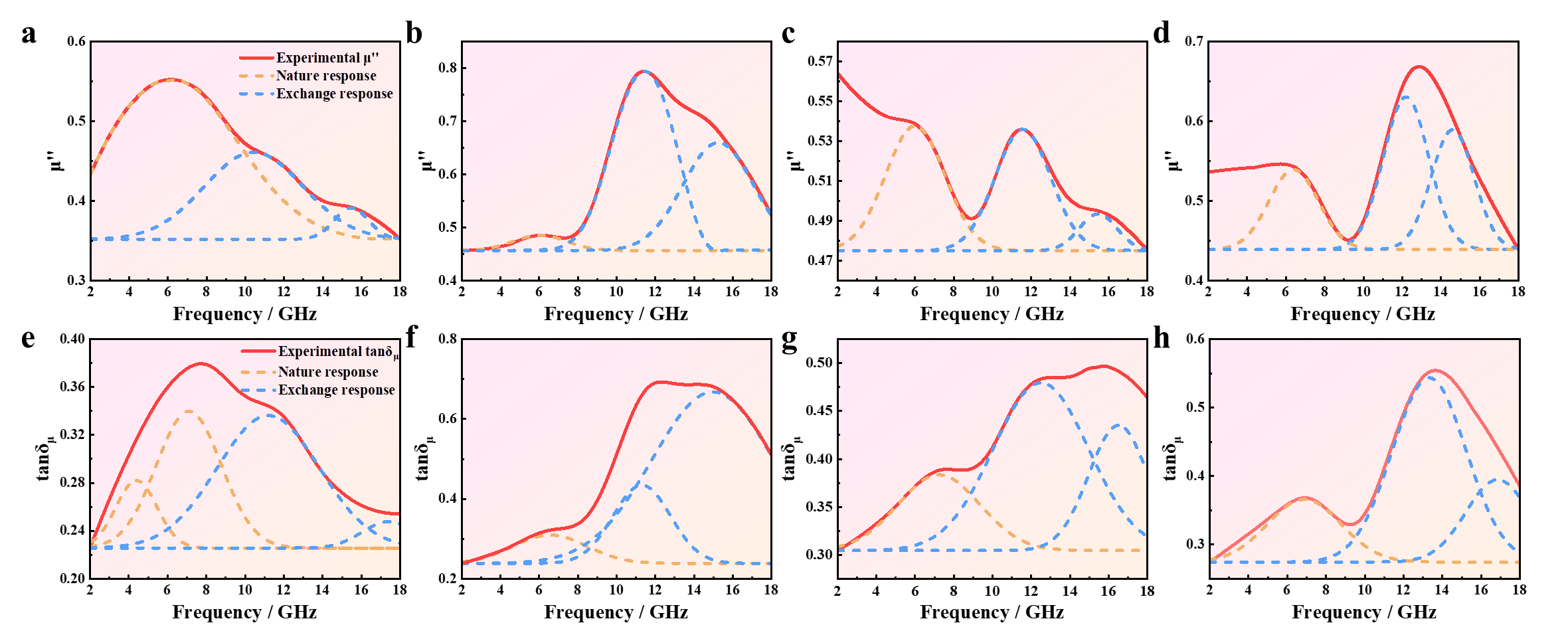


1. (a-d) Fitted curves of the imaginary part of the permeability of samples; (e-f) Fitted curves of the magnetic loss factor of samples

**
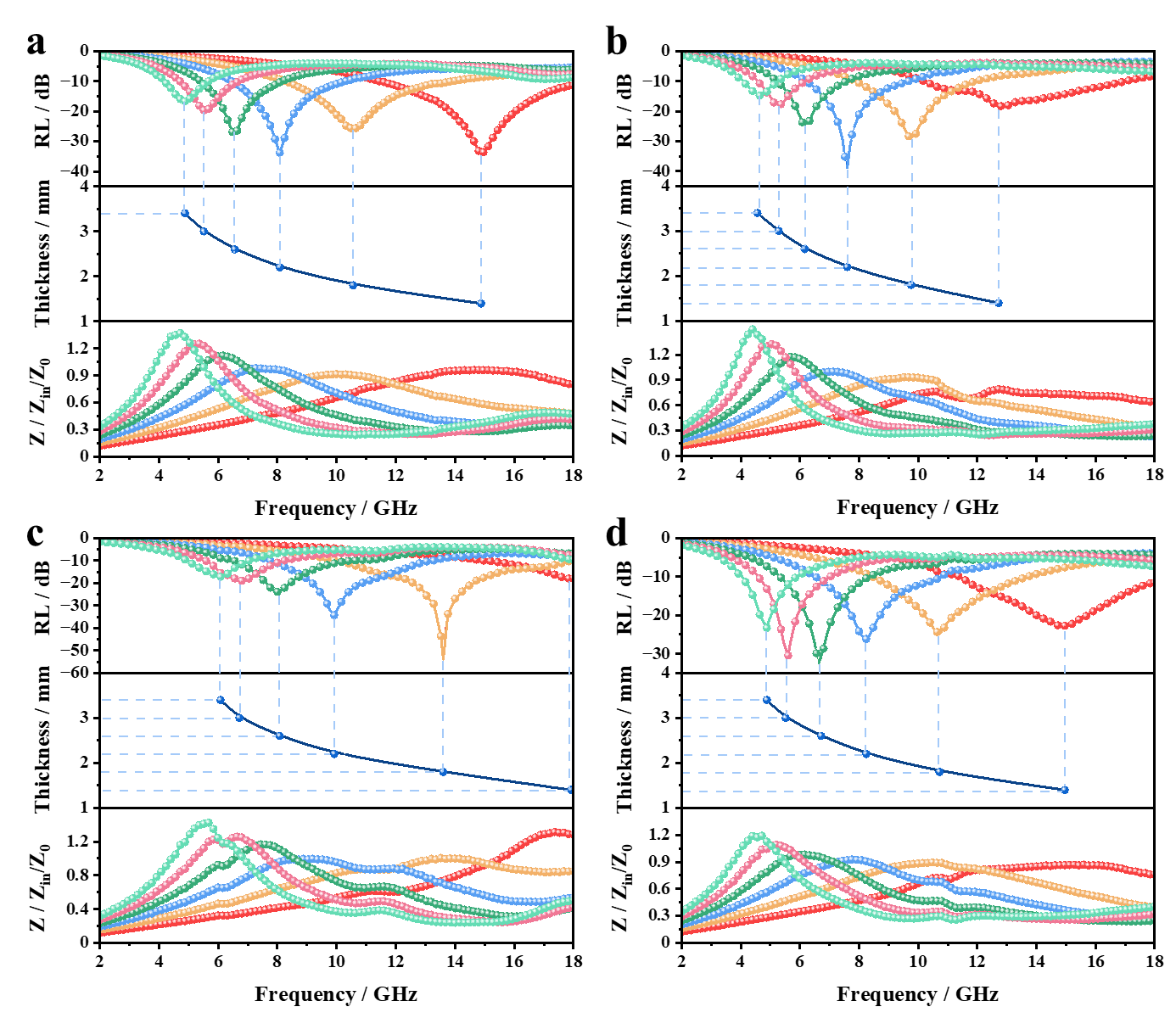
**

1. Impedance matching maps of (a) FCNC-0; (b) FCNC-1; (c) FCNC-2; (d) FCNC-3.
